# Hippocampal Mrp8/14 signaling plays a critical role in the manifestation of depressive-like behaviors in mice

**DOI:** 10.1186/s12974-018-1296-0

**Published:** 2018-09-04

**Authors:** Hong Gong, Wen-Jun Su, Zhi-Yong Cao, Yong-Jie Lian, Wei Peng, Yun-Zi Liu, Yi Zhang, Lin-Lin Liu, Ran Wu, Bo Wang, Ting Zhang, Yun-Xia Wang, Chun-Lei Jiang

**Affiliations:** 1 Department of Stress Medicine, Faculty of Psychology and Mental Health, Second Military Medical University, Shanghai, 200433 People’s Republic of China; 2 Hainan Branch of Chinese PLA General Hospital, Sanya, 572013 People’s Republic of China; 3 Department of Psychiatry, The 102nd Hospital of PLA, Changzhou, 213003 People’s Republic of China; 4 Department of Psychiatry, Faculty of Psychology and Mental Health, Second Military Medical University, Shanghai, 200433 People’s Republic of China; 5 Department of Navy Medicine, Second Military Medical University, Shanghai, 200433 People’s Republic of China

**Keywords:** Depression, Myeloid-related protein 8/14, TLR4, Stress, Neuroinflammation, Hippocampus

## Abstract

**Background:**

Depression is one of the most common mental disorders characterized mainly by low mood and loss of interest or pleasure. About a third of patients with depression do not respond to classic antidepressant treatments. Recent evidence suggests that Mrp8/14 (myeloid-related protein 8/14) plays a crucial role in cognitive dysfunction and neuroinflammatory diseases, yet its role in mood regulation remains largely uninvestigated. In the present work, we explored the potential role of Mrp8/14 in the progression of depression.

**Methods:**

After 4 weeks of chronic unpredictable mild stress (CUMS), depressive-like symptoms and Mrp8/14 were determined. To verify the effects of Mrp8/14 on depressive-like behaviors, the inhibitor TAK-242 and recombinant Mrp8/14 were used. Furthermore, the molecular mechanisms in Mrp8/14-induced behavioral and biological changes were examined in vivo and ex vivo.

**Results:**

Four-week CUMS contributed to the development of depressive symptoms. Mrp8 and Mrp14 were upregulated in the hippocampus and serum after exposure to CUMS. Pharmacological inhibition of Mrp14 attenuated CUMS-induced TLR4/NF-κB signaling activation and depressive-like behaviors. Furthermore, central administration of recombinant Mrp8, Mrp14, and Mrp8/14 resulted in neuroinflammation and depressive-like behaviors. Mrp8/14-provoked proinflammatory effects and depressive-like behaviors were improved by pretreatment with a TLR4 inhibitor. Moreover, pharmacological inhibition of TLR4 reduced the release of nitric oxide and reactive oxygen species in Mrp8/14-activated BV2 microglia.

**Conclusions:**

These data suggest that the hippocampal Mrp8/14-TLR4-mediated neuroinflammation contributes to the development of depressive-like behaviors. Targeting the Mrp8/14 may be a novel promising antidepressant approach.

**Electronic supplementary material:**

The online version of this article (10.1186/s12974-018-1296-0) contains supplementary material, which is available to authorized users.

## Background

Depression is a common mental disorder with high rates of recurrence [1]. Unfortunately, current main antidepressants, such as selective serotonin reuptake inhibitors (SSRIs) and tricyclic anti-depressants (TCAs), display unsatisfactory response rates and various side effects [2]. Although the etiology and pathophysiology of depression remain unknown, recent evidence suggests that inflammation can affect the brain and play a vital role in the psychopathology of depression [3, 4]. The previous reports from our and other laboratories have identified the underlying role of several endogenous alarmins or damage-associated molecular patterns (DAMPs), such as high mobility group box 1 (HMGB1) [5–7] and adenosine triphosphate (ATP) [8], in neuroinflammation and depressive symptoms.

Myeloid-related protein-8 (Mrp8, also termed S100A8) and myeloid-related protein-14 (Mrp14, also termed S100A9) are two important members of the Ca^2+^ binding S100 protein family. Mrp8 and Mrp14 have been identified as crucial endogenous DAMPs [9, 10]. However, compared with other DAMPs, less research has been conducted focusing on the effects of Mrp8 and Mrp14. Under physiological conditions, these two proteins exist and function mainly as a heterodimeric complex Mrp8/14 (also termed S100A8/S100A9 or calprotectin) [11, 12]. Mrp8/14 can be passively or actively released into the extracellular environment, and interacts with various membrane receptors, including Toll-like receptor 4 (TLR4), receptor for advanced glycation end products (RAGE) and CD36 [13, 14]. Then, the alarmin Mrp8/14 affects downstream signaling through NF-κB and plays a critical role in the inflammatory response. ABR-215757, the inhibitor of Mrp14, is currently assessed as a novel treatment for systemic lupus erythematosus (SLE) and systemic sclerosis [15]. Other evidence shows that Mrp8/14 plays vital roles in various inflammatory diseases, such as rheumatoid arthritis [16], psoriasis [14], shock [9], obesity [17], and cancer [18, 19].

In the central nervous systems (CNS), Mrp8 and Mrp14 have been found to be expressed in the microglia [20] and neurons [21, 22]. Mrp8 stimulation results in the induction of IL-1β and activation of NF-κB in astrocytes ex vivo [23]. Mrp8 and Mrp14 play key roles in several chronic or acute neurological disorders, including Alzheimer’s disease [20], epilepsy [23], meningitis [24], and CNS injury [25]. Recently, Stankiewicz and colleagues found that hippocampal Mrp8 and Mrp14 mRNA increased after chronic social stress [26], which could induce depressive-like behaviors in rodents. However, the specific role of Mrp8 and Mrp14 in neuroinflammation and depression is still undetected and far from clear.

In the present study, we investigated the levels of Mrp8 and Mrp14 expression in the hippocampus and serum after exposure to chronic unpredictable mild stress (CUMS). Further, the effects of Mrp14 inhibitor ABR-215757 on CUMS-induced neuroinflammation and depressive-like behaviors were assessed. Moreover, the recombinant proteins were injected into the cerebrospinal fluid (CSF) to determine the changes of depressive-like behaviors. Finally, the molecular mechanisms in Mrp8/14 heterodimer-induced behavioral and biological changes were examined in vivo and ex vivo.

## Methods

### Animals

Male BALB/c mice (6–8 weeks of age, 20–23 g of weight) were purchased from the Animal Center (Second Military Medical University, Shanghai, China). All animals were given 1–2 weeks to acclimate to colony conditions before the experiments began. The mice were maintained under standard conditions (humidity 52 ± 2%, temperature 22 ± 1 °C) with a 12-h light/dark cycle and ad libitum access to food and water unless otherwise stated. After acclimation, mice were randomly assigned to the control and experiment groups using random numbers generated in Microsoft Excel (Additional file 1: Figure S1). All experimental protocols were approved by the Second Military Medical University Animal Care Committee. All experiments were conducted in accordance with related guidelines and laws.

### Drugs and reagents

ABR-215757 (quinoline-3-carboxamide) was provided by Active Biotech AB (Lund, Sweden). Recombinant mouse Mrp8 (*E.coli* derived, purity > 95%, cat. no. ab108120) and Mrp14 (*E.coli* derived, purity > 90%, cat. no. ab109951) were purchased from Abcam (Abcam, Cambridge, UK). Recombinant mouse Mrp8/14 heterodimer (*E.coli* derived, purity > 95%, cat. no. 8916-S8) was obtained from R & D SYSTEMS (R & D SYSTEMS, Minneapolis, USA). The endotoxin level is less than 0.10 EU per 1 μg in the recombinant proteins. TAK-242 (C15H17CIFNO4S, cat. no. HY-11109) was obtained from MedChem Express (MedChem Express, New Jersey, USA). Anti-Mrp8 goat polyclonal antibody (cat. no. sc-8113), anti-Mrp14 goat polyclonal antibody (cat. no. sc-8115), anti-TLR4 mouse monoclonal antibody (cat. no. sc-293072), and anti-RAGE mouse monoclonal antibody (cat. no. sc-365154) were purchased from Santa Cruz. Anti-MyD88 rabbit monoclonal antibody (cat. no. 4283) and Anti- NF-κB p65 rabbit monoclonal antibody (cat. no. 8242) were obtained from Cell Signaling Technology (Danvers, MA, USA). Anti-Phospho-NF-κB p65 rabbit polyclonal antibody (cat. no. AB11011) was purchased from AbSci (Nanjing, China). Anti-iNOS rabbit monoclonal antibody (cat. no. ab205529) was purchased from Abcam. Anti-ACTB rabbit polyclonal antibody (cat. no. D110001–0100) was obtained from BBI Life Science. IRDye 800CW goat anti-mouse antibody (cat. no. 926–68070), goat anti-rabbit antibody (cat. no. 926–32211), and IRDye 680RD donkey anti-goat antibody (cat. no. 925–68074) were provided by LI-COR Biosciences.

### CUMS

As a classic animal model of depressive symptoms, CUMS model has been widely used for more than 20 years [27]. The CUMS protocol was performed as described previously [28]. Briefly, on a daily basis, mice were exposed to specific unpredictable stressors, including restraint for 2 h, cage shaking for 30 min, 45° cage tilt for 12 h, 4 °C swimming for 5 min, 45 °C oven for 10 min, damp bedding for 12 h, and food and water deprivation for 24 h. For the intervention experiment, ABR-215757 or normal saline was injected (IP) daily for the whole CUMS period. Generally, animals were housed in group cages (4–5 mice/cage) except during some of the manipulations (e.g., restraint stress) and tests (e.g., weighing and the sucrose preference test). The CUMS procedure generally lasts for 4 weeks. Body weight was assessed every week. Sucrose preference test and tail suspension test were adopted to identify the depressive-like behaviors.

### Behavioral manipulations and tests

Sucrose preference test (SPT) was performed as described in a previous study [29]. To determine sucrose preference, mice were provided with two bottles (randomized placement), filled with either tap water or 1% sucrose solution (*w*/*v*). Mice were acclimated to 1% sucrose for 48 h. The bottles were weighed both before and after the experiment, and consumption was quantified following overnight bottle choice. The sucrose preference was defined as the ratio of the weight of sucrose solution consumption to the total water intake, i.e., sucrose preference = sucrose consumption / (sucrose consumption + water consumption) × 100%. The test was performed in dark phase (6–10 p.m.).

Tail suspension test (TST) was carried out according to our previous report [30]. The mouse was suspended by the tail using adhesive tape for 1 min for acclimation and another 5 min for detection. The tail climbing behaviors were prevented by passing mouse tails through a small plastic cylinder prior to suspension. The immobility time during the latter 5-min-long suspension was recorded and analyzed by Tail Suspension SOF-821 (Med Associates, Inc., St. Albans, VT, USA). The experiment was conducted in the dark without interruption. To eliminate olfactory interference, the hooks and chambers were cleaned with 75% ethanol between two separated test sessions. Experimental grouping was blinded to the tester assessing immobility.

### Sample collection

After weighing and behavioral tests, mice under general anesthesia were fixed on a heated pad. Blood was collected from left ventricle. Then, mice were perfused transcardially with ice-cold saline for about 3 min. Hippocampi were isolated on ice, temporarily frozen in liquid nitrogen, and stored at − 80 °C. Blood samples were allowed to stand for 30 min at room temperature and centrifuged at 4000 rpm for 15 min. The supernatant serum was collected and stored at − 80 °C. Cell samples were prepared as stated below.

### Enzyme-linked immunosorbent assay

Mouse serum Mrp8 and Mrp14 were determined using enzyme-linked immunosorbent assay kits (ELISAs, cat. no. CSB-EL020641MO and CSB-EL020642MO) from CUSABIO and CusAb (Wuhan, China). All the assays were performed according to the manufacturer’s instructions. The detection range was 0.625–40 ng/mL for Mrp8, 0.45–30 ng/mL for Mrp14. The ELISA kits also have high precision, including high intra-assay precision (CV < 8%) and inter-assay precision (CV < 10%).

### Western blot

The frozen mouse hippocampi and BV2 microglia were homogenized in ice-cold RIPA buffer (Beyotime Institute of Biotechnology, Nantong, Jiangsu, China) containing 1 mM protease inhibitor PMSF (Beyotime Institute of Biotechnology) and 10% PhosSTOP phosphatase inhibitor (Roche, Indianapolis, IN, USA). Protein concentration in the lysate was determined with the BCA assay (Beyotime Institute of Biotechnology). Then, samples were mixed with 5× loading buffer and heated at 100 °C for 10 min. Protein samples were separated by 10–15% sodium dodecyl sulfate-polyacrylamide (SDS-PAGE) gel electrophoresis and transferred to polyvinylidene difluoride membranes (Millipore, Billerica, USA). After two-hour blocking with 5% nonfat milk at room temperature, membranes were incubated with primary antibodies overnight at 4 °C. After washing, membranes were further incubated with fluorescent second antibodies for 1 h at room temperature. Bands were visualized using Odyssey Infrared Imaging System (LI-COR, Inc., Lincoln, NE, USA) and quantified with National Institutes of Health (NIH) Image J software.

### Stereotaxic surgery

Intracerebroventricular (ICV) cannulation was operated for ICV injection as described previously [6]. Briefly, after anesthesia, the dorsal aspect of the skull was shaved and swabbed with 75% ethanol. The mice were subsequently fixed on a stereotaxic apparatus (RWD Life Science, Shenzhen, China). A guide cannula was vertically implanted in the right ventricle according to the following stereotaxic coordinates: anteroposterior − 0.6 mm; mediolateral − 1.1 mm; and dorsal ventricular − 2 mm. Then, a matched syringe needle was placed into the cannula, and the syringe was removed at 3 min after injection. To verify entry into the right ventricle before our main experiments, 5 μL of trypan blue dye was injected into the cannula, and the mouse brain was cut into slices for observing. Before the initiation of following experiments, animals were allowed 2 weeks to recover after operations.

### Drug administration for animals

Mrp14 inhibitor ABR-215757 was dissolved and prepared as recommended by Active Biotech. Other drugs were dissolved or diluted using sterile endotoxin-free isotonic saline. ABR-215757 (10 mg/kg body weight [31]) was administrated through intraperitoneal (IP) injection once a day during CUMS period. Recombinant mouse Mrp8 (3 μg, 6 μL [32]), Mrp14 (3 μg, 6 μL), and Mrp8/14 heterodimer (3 μg, 6 μL) were injected via ICV administration. Recombinant proteins were diluted using normal saline for ICV injection. The control mice were treated with 6 μL normal saline (Additional file 1: Figure S1). This dose was selected based on a previous report [32], which showed recombinant Mrp8 promoted TLR4 activation in PBMCs and microglia. Behavioral tests were conducted 24 h after the recombinant protein treatment. TLR4 inhibitor TAK-242 was injected (IP, 3 mg/kg body weight [6]) 30 min prior to rMrp8/14 administration (Additional file 1: Figure S1). These inhibitors (ABR-215757 and TAK-242) can permeate the blood–brain barrier [33].

### Real-time RT-PCR

The levels of gene expression of inflammatory cytokines were determined by real-time RT-PCR. Brain tissue was stored in liquid nitrogen, and BV2 microglia were washed twice with ice cold PBS and then processed for RNA extraction. Total RNA was extracted from the hippocampus or BV2 microglia utilizing a standard method of TRIzol reagent (Invitrogen, Carlsbad, CA, USA). After quantification, 2 μg of total RNA was used for cDNA synthesis using PrimeScript™ RT Master Mix (TaKaRa, Shiga, Japan). Primer sequences were tested for sequence specificity using Primer-BLAST in NCBI. As tabulated in Additional file 2: Table S1, the primers used in this study were obtained commercially from Sangon Biotechnology (Shanghai, China). The RT-PCR amplification was performed using 2 μL of cDNA and MaximaTM SYBR Green/ROX qPCR Master Mix (Fermentas, Waltham, MA, USA) on an Applied Biosystems 7500 (Life Technologies Corporation., Carlsbad, CA, USA). Melting curve analysis was utilized to verify primer specificity, and a comparative threshold cycle method was adopted to determine the fold changes of each gene expression relative to β-actin.

### Cell culture and treatment

A murine microglial cell line BV2 was obtained from American Type Culture Collection (ATCC, Rockville, MD, USA). Cells were maintained in Dulbecco’s modified Eagle’s medium (DMEM; Life Technologies/Gibco, Grand Island, NY, USA) supplemented with 10% fetal bovine serum (FBS, Life Technologies/Gibco) at 37 °C in an incubator with 95% air and 5% CO_2_. For nitric oxide (NO) assay and Western Blot, cells were seeded onto 6-well culture plates. For real-time RT-PCR and reactive oxygen species (ROS) assay, cells were seeded onto 12-well culture plates. After treatment with TAK-242 (1 μM) for 30 min, cells were administrated with rMrp8/14 (0.5 μg/mL) for 24 h. Then, the supernatants were collected for NO assay, and cells were used for Western Blot, real-time RT-PCR, and ROS assay.

### Determinations of NO and ROS

NO of cell supernatant was detected using Griess Reagent (Beyotime Institute of Biotechnology, Haimen, China) according to the manufacturer’s instructions. Samples and standards were added into a 96-well plate, and then Griess Reagent I and II were added successively. The absorbance was determined at a wavelength of 540 nm using a microplate reader. ROS was determined by a Reactive Oxygen Species Assay Kit (Beyotime Institute of Biotechnology). In brief, after washing, cells were incubated with fluorescent probe DCFH-DA (10 μM, Beyotime Institute of Biotechnology) for 20 min at 37 °C in an incubator. Then, the extracellular DCFH-DA was cleared, and images were obtained using a fluorescence microscope (Carl Zeiss) at the 488 nm excitation wavelength and 525 nm emission wavelength.

### Statistical analysis

All data were presented as mean ± standard error of the mean (SEM). Normality and homogeneity of variance were assessed before comparisons using Kolmogorov–Smirnov test and Levene’s test. Statistical analyses were carried out with Student’s *t* test when comparing between two variables, and one-way or two-way ANOVA followed by LSD post hoc tests when comparing among multiple variables. Omnibus *F* values with degrees of freedom were reported for each ANOVA. Differences were considered statistically significant only when *p* < 0.05. All the analyses were performed with IBM SPSS 21.0 (IBM Corp., Armonk, N.Y.).

## Results

### CUMS-induced depressive-like behaviors associated with the increases of Mrp8 and Mrp14 in the hippocampus and serum

**Fig. 1:**
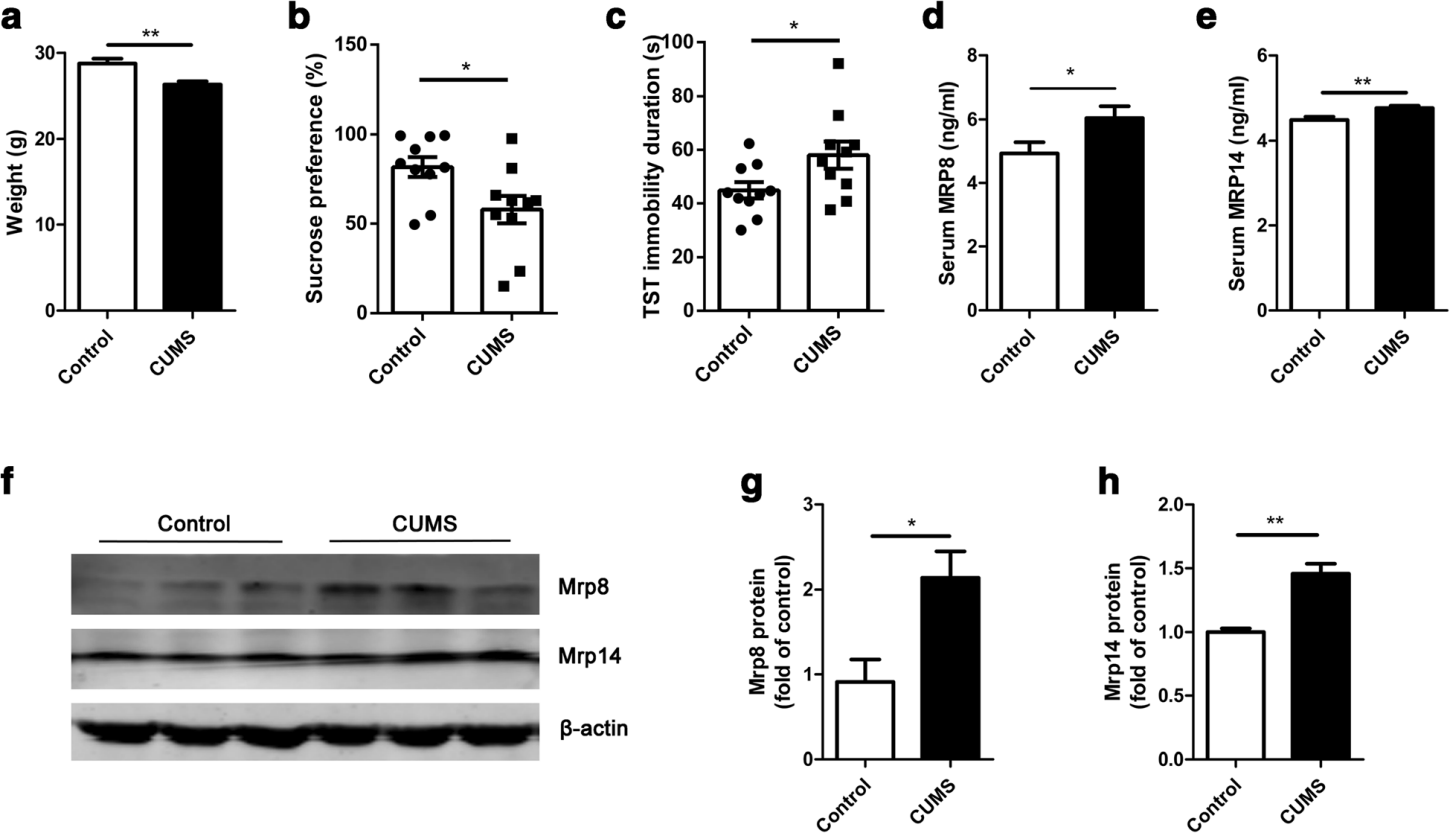
CUMS induced depressive-like phenotypes and upregulated the expression of Mrp8 and Mrp14 in the hippocampus and serum. **a** After 4-week chronic unpredictable mild stress (CUMS), mice had a lower body weight than control mice (*n* = 14–15 mice/group). **b, c** CUMS resulted in depressive-like behavior, including decreased sucrose preference (*n* = 10 mice/group) and increased immobility time in tail suspension test (TST) (*n* = 10 mice/group). **d, e** Protein levels of Mrp8 (**d**) and Mrp14 (**e**) were slightly but statistically significantly increased in the serum of CUMS-exposed mice (*n* = 10–11 mice/group). **f** Representative images of western blot. Proteins were extracted from the hippocampus. **g, h** The quantitative analyses revealed that both Mrp8 (*n* = 3 mice/group) and Mrp14 (*n* = 3 mice/group) were increased in the hippocampus of stress mice. *p < 0.05, **p < 0.01 between two groups

Behavioral tests and body weight were measured after CUMS period (Additional file 1: Figure S1a). Coinciding with previous studies [6, 27], 4-week CUMS could induce the depressive-like behaviors in rodents. Specifically, the body weight was significantly attenuated in CUMS-exposed mice compared with control mice (Fig. 1a; *t*_(27)_ = 3.63, *p* < 0.01, 29.18 ± 0.53 vs. 25.78 ± 0.41). CUMS significantly decreased the sucrose preference, indicating the impaired sensitivity to reward and anhedonia, which is a core symptom of major depression (Fig. 1b; *t*_(18)_ = 2.51, *p* = 0.02, 81.65 ± 5.57 vs. 57.87 ± 7.66). The stressed mice also showed longer immobility duration in the TST (Fig. 1c; *t*_(18)_ = 2.22, *p* = 0.04, 44.87 ± 3.05 vs. 57.98 ± 5.05). On the other hand, the results of ELISA showed that relative to the control group, serum Mrp8 (Fig. 1d; *t*_(20)_ = 2.15, *p* = 0.04, 4.93 ± 0.35 vs. 6.04 ± 0.37) and Mrp14 (Fig. 1e; *t*_(20)_ = 2.96, *p* < 0.01, 4.49 ± 0.08 vs. 4.77 ± 0.06) were slightly upregulated after CUMS intervention. Western blot revealed that hippocampal Mrp8 (Fig. 1f and g; *t*_(4)_ = 3.00, *p* = 0.01, 0.91 ± 0.26 vs. 2.14 ± 0.31) and Mrp14 (Fig. 1f and h; *t*_(4)_ = 5.41, *p* < 0.01, 1.10 ± 0.03 vs. 1.46 ± 0.08) were also increased. Collectively, these data demonstrate that CUMS elicited depressive-like behaviors and increased Mrp8 and Mrp14 protein in both serum and hippocampus.

### CUMS activated TLR4 signaling pathway but did not affect RAGE protein expression

**Fig. 2:**
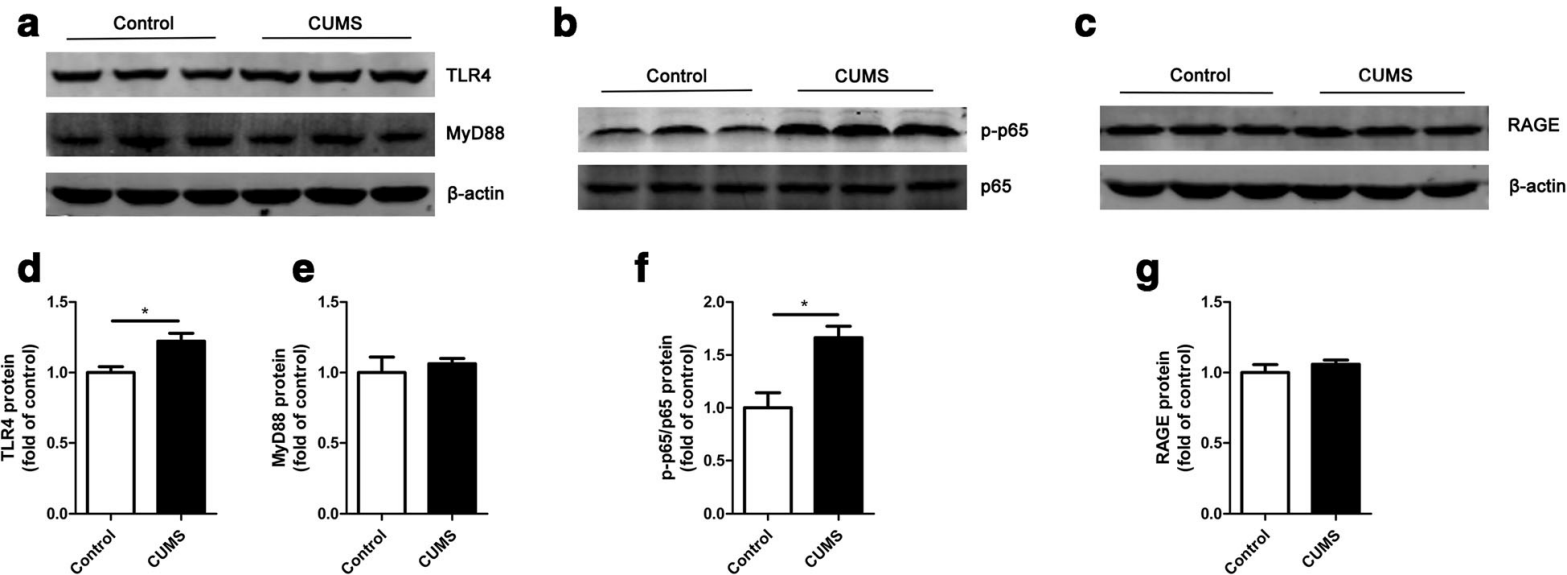
CUMS activated TLR4 instead of RAGE signaling pathway. **a, d, e** After 4-week CUMS, the expression of hippocampal TLR4 was increased (**d**), while MyD88 did not change significantly (**e**). **b, f** CUMS activated the phosphorylation of hippocampal NF-κB p65. **c, g** No significant difference of RAGE expression was found between the control group and stress group. *n* = 3 mice/group, *p < 0.05 between two groups

TLR4 signaling pathway has been shown to have a pivotal role in stress-induced neuroinflammation [34] and major depressive disorder [35]. To validate whether TLR4 signaling pathway is activated in the hippocampus of stressed mice, we detected the protein level of TLR4, MyD88, and the NF-κB p65 phosphorylation. Stress exposure increased TLR4 (Fig. 2a and d; *t*_(4)_ = 3.18, *p* = 0.03, 1.00 ± 0.04 vs. 1.22 ± 0.06) but not MyD88 (Fig. 2a and e; *t*_(4)_ = 0.53, *p* = 0.62, 1.00 ± 0.11 vs. 1.06 ± 0.04) at the protein level. CUMS also activated the phosphorylation of NF-κB p65 (Fig. 2b and f; *t*_(4)_ = 3.74, *p* = 0.02, 1.00 ± 0.14 vs. 1.66 ± 0.11). Conversely, RAGE expression remained unaffected (Fig. 2c and g; *t*_(4)_ = 0.92, *p* = 0.41, 1.00 ± 0.06 vs. 1.06 ± 0.03). Taken together, CUMS activated TLR4/NF-κB signaling pathway but did not affect the expression of RAGE.

### ABR-215757 improved depressive-like behaviors and inhibited TLR4/NF-κB signaling activation induced by CUMS

**Fig. 3:**
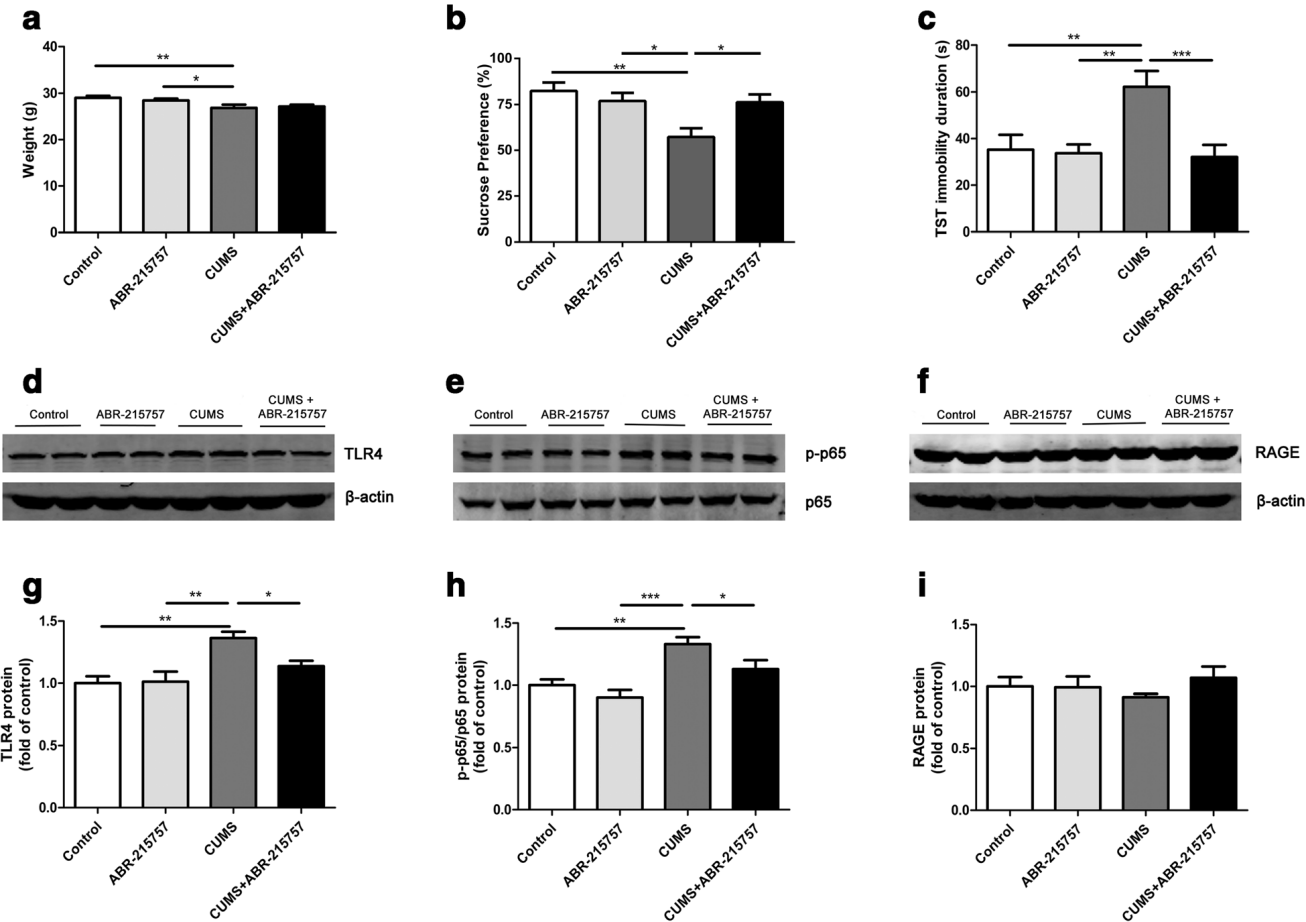
Mrp14 inhibitor (ABR-215757) attenuated depressive-like behavior and TLR4/NF-κB signaling pathway activation induced by CUMS. ABR-215757 and normal saline were injected (IP) daily for the whole CUMS period. **a** The body weight of CUMS-treated mice was markedly reduced compared with control group or ABR-215757 group, whereas no significant differences were observed between CUMS + ABR-215757 group and any other group (*n* = 6–10 mice/group). **b, c** CUMS + ABR-215757 group displayed higher sucrose preference (*n* = 10 mice/group) and less immobility duration in tail suspension test (*n* = 6–9 mice/group). **d**–**i** Western blot analyses showed the changes of hippocampal TLR4/NF-κB signaling and RAGE protein (*n* = 4 mice/group). *p < 0.05, **p < 0.01, ***p < 0.001 between two indicated groups

To further investigate the functional role of Mrp14 in the development of depressive symptoms, the inhibitor ABR-215757 was injected daily during the CUMS period (Additional file 1: Figure S1b). As shown in Fig. 3a, the body weight of mice in CUMS group was significantly lower than control or vehicle mice (*F*_(1,29)_ = 4.54, *p* = 0.01; post hoc: vehicle vs. CUMS, *p* = 0.03, 28.44 ± 1.29 vs. 26.84 ± 1.78), while CUMS could not significantly decrease body weight of ABR-215757-treated mice (post hoc: ABR-215757 vs. CUMS + ABR-215757, *p* = 0.06, 28.44 ± 1.29 vs. 27.16 ± 1.08). However, ABR-215757 did not significantly rescue body weight of stressed mice (post hoc: CUMS vs. CUMS + ABR-215757, *p* = 0.66, 26.84 ± 1.78 vs. 27.16 ± 1.08). Besides, ABR-215757 increased the sucrose preference (Fig. 3b; *F*_(1,36)_ = 5.67, *p* = 0.02; post hoc: CUMS vs. CUMS + ABR-215757, *p* < 0.05, 57.28 ± 4.79 vs. 76.31 ± 4.26) and decreased the immobility duration in TST (Fig. 3c; *F*_(1,26)_ = 6.91, *p* < 0.01; post hoc: CUMS vs. CUMS + ABR-215757, *p* < 0.001, 62.21 ± 6.70 vs. 32.12 ± 5.15). We also found that ABR-215757 alleviated TLR4 expression (Fig. 3d and g; *F*_(1,12)_ = 7.85, *p* < 0.01; post hoc: CUMS vs. CUMS + ABR-215757, *p* = 0.02, 1.36 ± 0.05 vs. 1.14 ± 0.04) and p65 phosphorylation (Fig. 3e and h; *F*_(1,12)_ = 9.68, *p* < 0.01; post hoc: CUMS vs. CUMS + ABR-215757, *p* = 0.03, 1.33 ± 0.06 vs. 1.13 ± 0.07) after CUMS exposure. The RAGE expression did not change significantly among these groups (Fig. 3f and i; *F*_(1,12)_ = 0.72, *p* = 0.56, 0.91 ± 0.03 vs. 1.07 ± 0.09). Thus, ABR-215757 could alleviate CUMS-induced depressive-like behaviors and TLR4/NF-κB signaling activation.

### Central injection of Mrp8, Mrp14, and Mrp8/14 induced depressive-like behaviors, proinflammatory cytokines production, and TLR4/NF-κB signaling activation

**Fig. 4:**
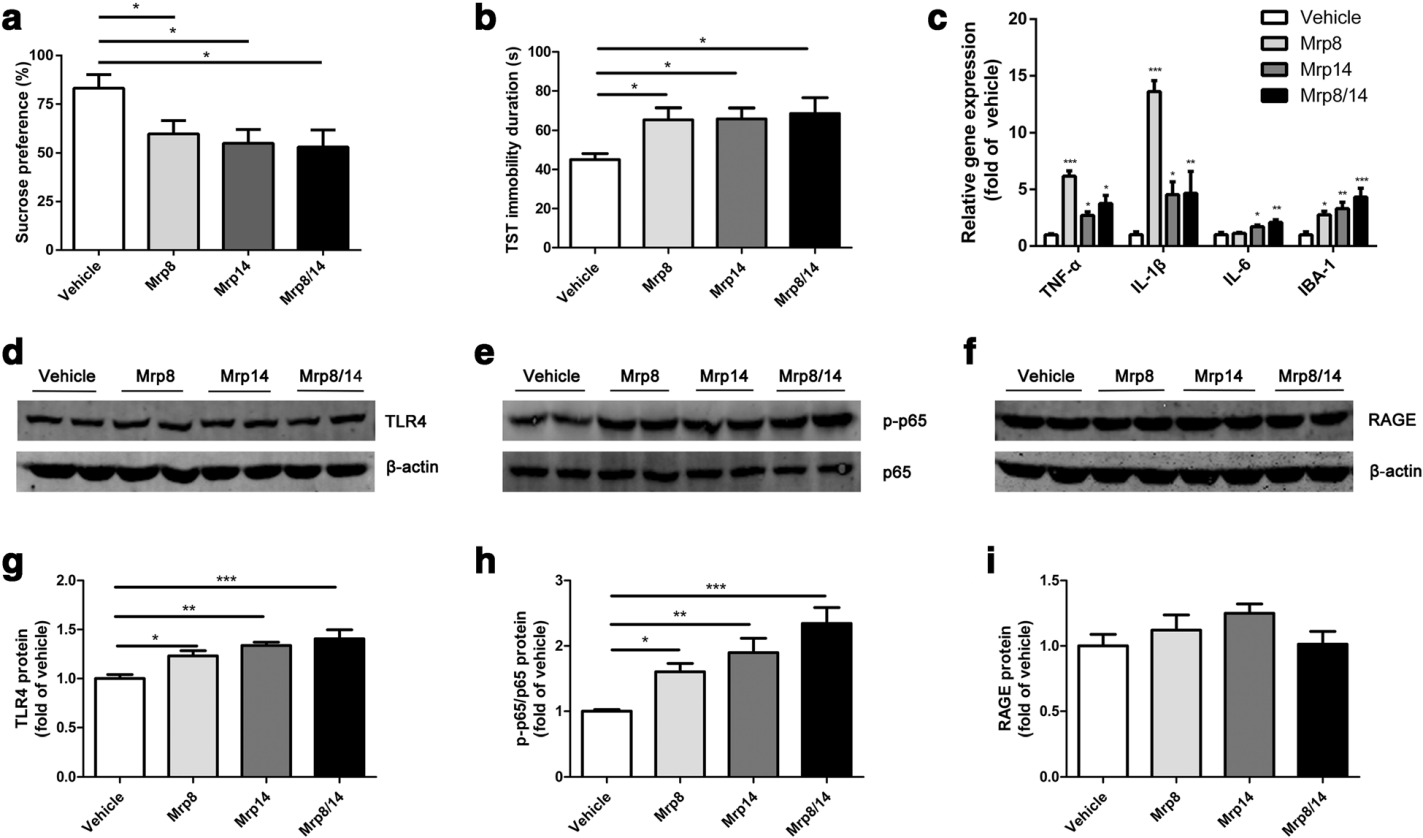
Changes in behavioral performance, cytokine expression, and TLR4/NF-κB signaling in response to recombinant proteins. After treatment with recombinant Mrp8, Mrp14, or Mrp8/14, depressive-like behaviors were determined by the sucrose preference test and tail suspension test (TST). **a** Both Mrp14- and Mrp8/14-treated mice exhibited reduced sucrose preference compared with the vehicle group (*n* = 10 mice/group). **b** All of the three recombinant proteins could extend the immobility time in TST compared with vehicle treatment (*n* = 5–6 mice/group). **c** The results of RT-PCR of the mRNA changes of proinflammatory cytokines and IBA-1 in the hippocampus. *n* = 4–5 mice/group for TNF-α, IL-6, and IBA-1, *n* = 3–5 mice/group for IL-1β. **d**–**i** Western blot analyses showed that hippocampal TLR4/NF-κB signaling was activated after these protein treatments, while the RAGE protein was not significantly changed among these groups (*n* = 4 mice/group). *p < 0.05, **p < 0.01, ***p < 0.001 between two indicated groups. The data are presented as the mean ± SEM

To determine whether Mrp8, Mrp14, and Mrp8/14 affected the development of depressive-like behaviors, recombinant Mrp8, Mrp14, Mrp8/14, and vehicle reagent were injected (ICV) to mice (Additional file 1: Figure S1c). Mice administrated with Mrp14 and Mrp8/14 presented lower sucrose preference (Fig. 4a; *F*_(3,36)_ = 3.44, *p* = 0.03; post hoc: vehicle vs. Mrp8, Mrp14, and Mrp8/14, all *p* < 0.05, 83.17 ± 7.02 vs. 59.90 ± 6.83, 55.03 ± 7.08, 53.09 ± 8.82). All of the recombinant Mrp8, Mrp14, and Mrp8/14 treatment could result in a longer immobility duration (Fig. 4b; *F*_(3,18)_ = 3.16, *p* = 0.05; post hoc: vehicle vs. Mrp8, *p* = 0.03; vehicle vs. Mrp14, *p* = 0.02; vehicle vs. Mrp8/14, *p* = 0.02, 45.02 ± 3.11 vs. 65.40 ± 6.08, 65.91 ± 5.45, 68.61 ± 7.92). Besides, we found that the mRNA levels of inflammatory cytokines (TNF-α, IL-1β, IL-6) were elevated in the hippocampus after being challenged by any of these recombinant proteins (Fig 4c; *F*_(3,14)_ = 20.54, *p* < 0.001 for TNF-α, 1.00 ± 0.10 vs. 6.17 ± 0.47, 2.70 ± 0.31, 3.76 ± 0.71; *F*_(3,14)_ = 24.70, *p* < 0.001 for IL-1β, 1.00 ± 0.26 vs. 13.60 ± 0.97, 4.54 ± 1.14, 4.67 ± 1.93; *F*_(3,14)_ = 7.59, *p* < 0.01 for IL-6, 1.00 ± 0.22 vs. 1.13 ± 0.08, 1.72 ± 0.14, 2.09 ± 0.23). Microglia activation has been suggested to be mainly responsible for depression [36, 37]. The results of RT-PCR indicated that recombinant proteins could upregulate IBA-1 gene expression (Fig. 4c; *F*_(3,14)_ = 7.79, *p* < 0.01; post hoc: vehicle vs. Mrp8, *p* = 0.03; vehicle vs. Mrp14, *p* < 0.01; vehicle vs. Mrp8/14, *p* < 0.001, 1.00 ± 0.25 vs. 2.76 ± 0.31, 3.30 ± 0.57, 4.85 ± 0.79). After these recombinant proteins treatment, the hippocampal TLR4 level (Fig. 4d and g; *F*_(3,12)_ = 9.13, *p* < 0.01; post hoc: vehicle vs. Mrp8, *p* = 0.02; vehicle vs. Mrp14, *p* < 0.01; vehicle vs. Mrp8/14, *p* < 0.001, 1.00 ± 0.04 vs. 1.23 ± 0.05, 1.34 ± 0.03, 1.41 ± 0.09) and p65 phosphorylation (Fig. 4e and h; *F*_(3,12)_ = 10.29, *p* < 0.01; post hoc: vehicle vs. Mrp8, *p* = 0.03; vehicle vs. Mrp14, *p* < 0.01; vehicle vs. Mrp8/14, *p* < 0.001, 1.00 ± 0.03 vs. 1.60 ± 0.13, 1.90 ± 0.22, 2.34 ± 0.24) were elevated. No significant differences were found in RAGE expression among the four groups (Fig. 4f and i; *F*_(3,12)_ = 1.51, *p* = 0.26, *p* < 0.001, 1.00 ± 0.09 vs. 1.12 ± 0.12, 1.25 ± 0.07, 1.01 ± 0.10). Collectively, central administration of Mrp8, Mrp14, and Mrp8/14 induced depressive-like behaviors, enhanced TLR4/NF-κB signaling pathway, and promoted the generation of proinflammatory cytokines.

### TAK-242 attenuated Mrp8/14-induced depressive-like behaviors, p65 phosphorylation, and proinflammatory cytokines production in vivo

**Fig. 5:**
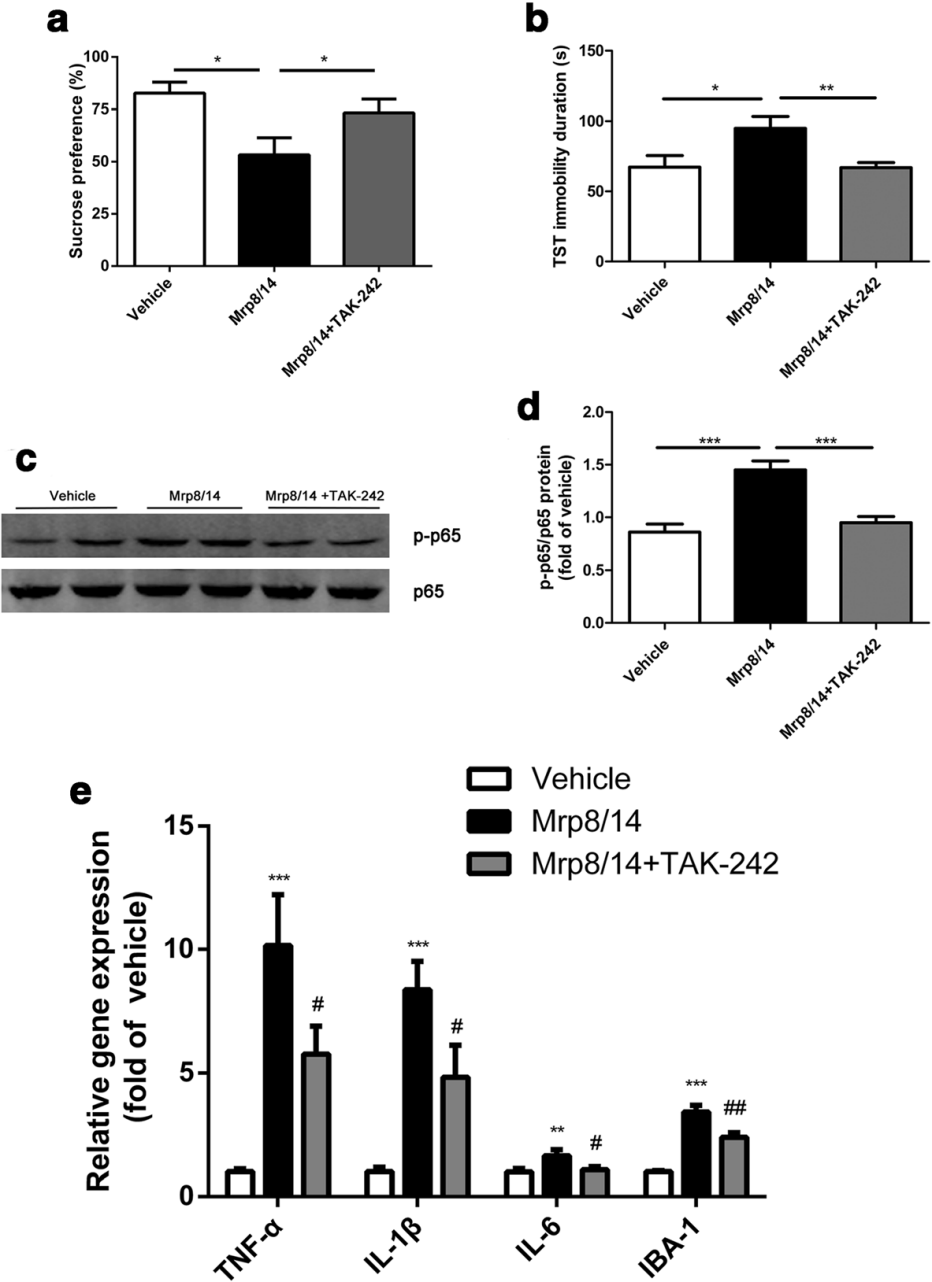
The effects of TAK-242 on depressive-like behaviors, and proinflammatory cytokine mRNA expression evoked by Mrp8/14. TAK-242 was administrated (IP) 30 min before the ICV injection of the Mrp8/14 heterodimer. **a**, **b** TAK-242 could alleviate the depressive-like behavior induced by Mrp8/14 (*n* = 7–10 mice/group). **c**, **d** The phosphorylation of NF-κB p65 evoked by Mrp14 was decreased after TAK-242 treatment (*n* = 4 mice/group). **e** The mRNA expression of proinflammatory cytokines and IBA-1 induced by Mrp8/14 was reduced by TAK-242 administration (*n* = 6–8 mice/group). *p < 0.05, **p < 0.01, ***p < 0.001 vs vehicle group. ^#^*p* < 0.05, ^##^*p* < 0.01 vs Mrp8/14 group

Since Mrp8/14 heterodimer is the most abundant form [11, 12], we conducted further studies in vivo and ex vivo using Mrp8/14 instead of Mrp8 and Mrp14. To elucidate whether TLR4 was a mediator in Mrp8/14-evoked depressive-like behaviors, mice were pretreated with TLR4 inhibitor TAK-242 before the ICV injection of recombinant Mrp8/14 (Additional file 1: Figure S1d). As expected, compared with the recombinant Mrp8/14-group mice, the recombinant Mrp8/14 + TAK-242-group mice had significantly higher sucrose preference (Fig. 5a; *F*_(2,23)_ = 4.99, *p* = 0.01; post hoc: Mrp8/14 vs. Mrp8/14 + TAK-242, *p* < 0.01, 53.30 ± 8.19 vs. 73.40 ± 6.48) and shorter immobility time in the tail suspension test (Fig. 5b; *F*_(2,23)_ = 5.24, *p* = 0.01; post hoc: Mrp8/14 vs. Mrp8/14 + TAK-242, *p* < 0.01, 94.91 ± 8.56 vs. 66.88 ± 3.69). The NF-κB p65 phosphorylation evoked by recombinant Mrp8/14 was inhibited by TAK-242 treatment (Fig. 5c and d; *F*_(2,9)_ = 18.21, *p* < 0.001; post hoc: Mrp8/14 vs. Mrp8/14 + TAK-242, *p* < 0.001, 1.45 ± 0.09 vs. 0.95 ± 0.06). We further detected the changes of the expression of proinflammatory cytokines. The results showed that relative to the recombinant Mrp8/14 group, the mRNA levels of proinflammatory cytokines in the hippocampus of recombinant Mrp8/14 + TAK-242 were reduced significantly (Fig. 5e; post hoc: Mrp8/14 vs. Mrp8/14 + TAK-242, all *p* < 0.05; 10.16 ± 2.06 vs. 5.77 ± 1.14 for TNF-α, 10.16 ± 1.15 vs. 4.83 ± 1.30 for IL-1β, 1.65 ± 0.24 vs. 1.08 ± 0.13 for IL-6). Moreover, Mrp8/14-induced hippocampal IBA-1 overexpression was attenuated by TAK-242, which was shown by the RT-PCR assay of IBA-1 (Fig. 5e; *F*_(2,20)_ = 43.71, *p* < 0.001; post hoc: Mrp8/14 vs. Mrp8/14 + TAK-242, *p* < 0.01, 3.42 ± 0.27 vs. 2.40 ± 0.19). Taken together, pretreatment of TAK-242 could inhibit the Mrp8/14-induced depressive-like behaviors and neuroinflammation.

### TAK-242 attenuated Mrp8/14-induced microglia activation and inflammatory cytokines production ex vivo

**Fig. 6:**
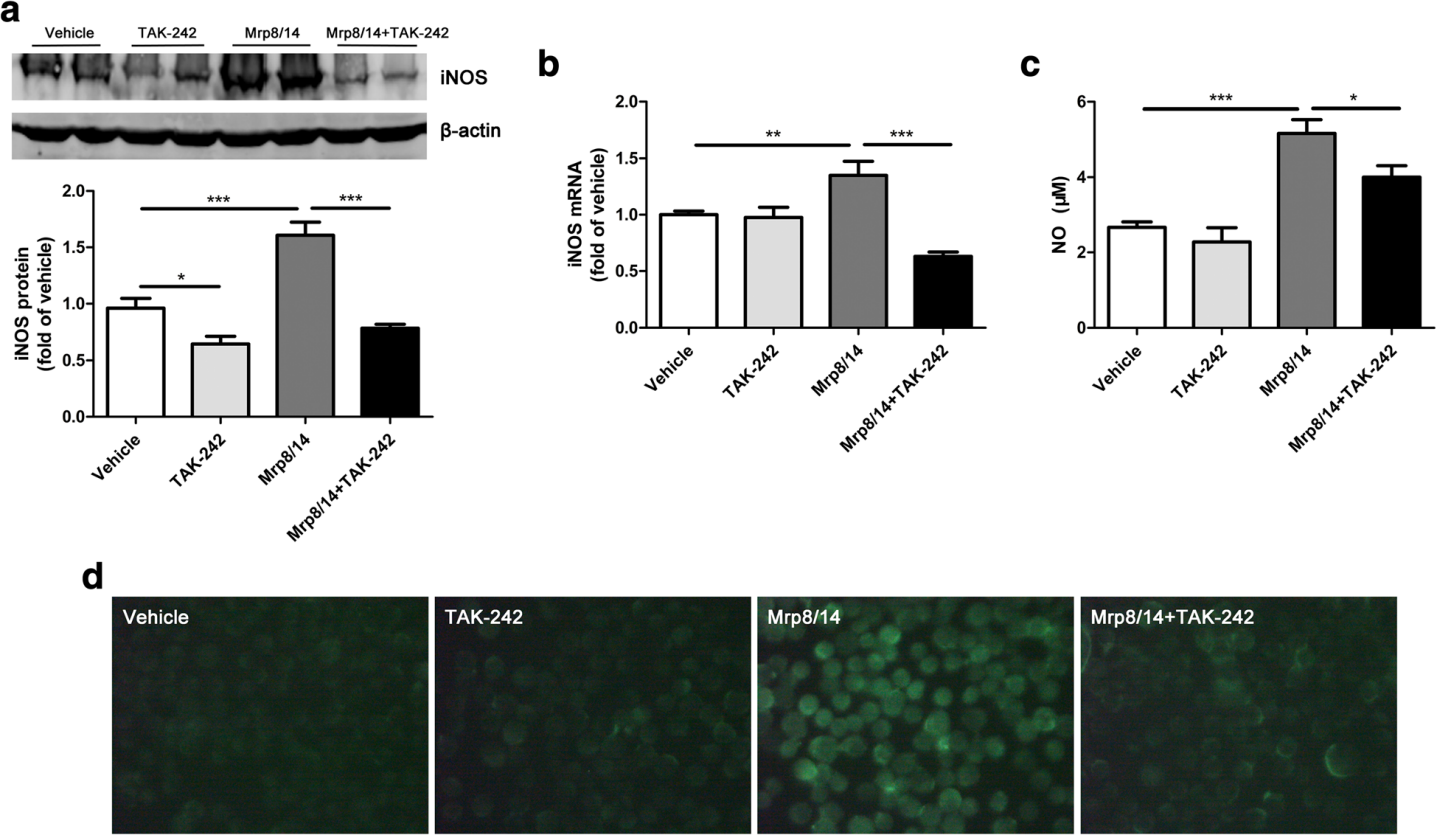
Mrp8/14-induced BV2 microglia activation was alleviated by a TLR4 inhibitor (TAK-242). Cells were administrated with TAK-242 for 30 min before treatment of Mrp8/14 heterodimer. **a** The iNOS protein level was reduced in Mrp8/14 + TAK-242 group compared with Mrp8/14 group (*n* = 4 per group). **b** The iNOS mRNA level was reduced in Mrp8/14 + TAK-242 group compared with Mrp8/14 group (*n* = 3–4 per group). **c** The production of nitric oxide (NO) induced by Mrp8/14 was decreased when pretreatment of TAK-242 (*n* = 3–4 per group). **d** The representative photos exhibited that the production of reactive oxygen species (ROS) induced by Mrp8/14 was reduced when pretreatment of TAK-242. *p < 0.05, **p < 0.01, ***p < 0.001 between two indicated groups

To further verify the effects of TAK-242 on recombinant Mrp8/14-induced microglia activation, we performed experiments using BV2 microglia. Two of the most important molecules released by activated microglia are the bioactive free radical NO [38] and ROS [39]. The increase of iNOS expression in both protein level and mRNA level was attenuated by pretreatment of TAK-242 in BV2 cells (Fig. 6a; *F*_(1,12)_ = 26.66, *p* < 0.001; post hoc: Mrp8/14 vs. Mrp8/14 + TAK-242, *p* < 0.001, 1.60 ± 0.12 vs. 0.78 ± 0.04; Fig. 6b; *F*_(1,11)_ = 14.19, *p* < 0.001; post hoc: Mrp8/14 vs. Mrp8/14 + TAK-242, *p* < 0.001, 1.34 ± 0.13 vs. 0.63 ± 0.04). The production of NO was also significantly decreased after TAK-242 administration (Fig. 6c; *F*_(1,11)_ = 17.81, *p* < 0.01; post hoc: Mrp8/14 vs. Mrp8/14 + TAK-242, *p* = 0.03, 5.17 ± 0.36 vs. 4.00 ± 0.30). Moreover, our data showed that TAK-242 reduced the generation of ROS induced by recombinant Mrp8/14 (Fig. 6d). Thus, Mrp8/14-induced microglia activation was mediated by TLR4.

## Discussion

**Fig. 7:**
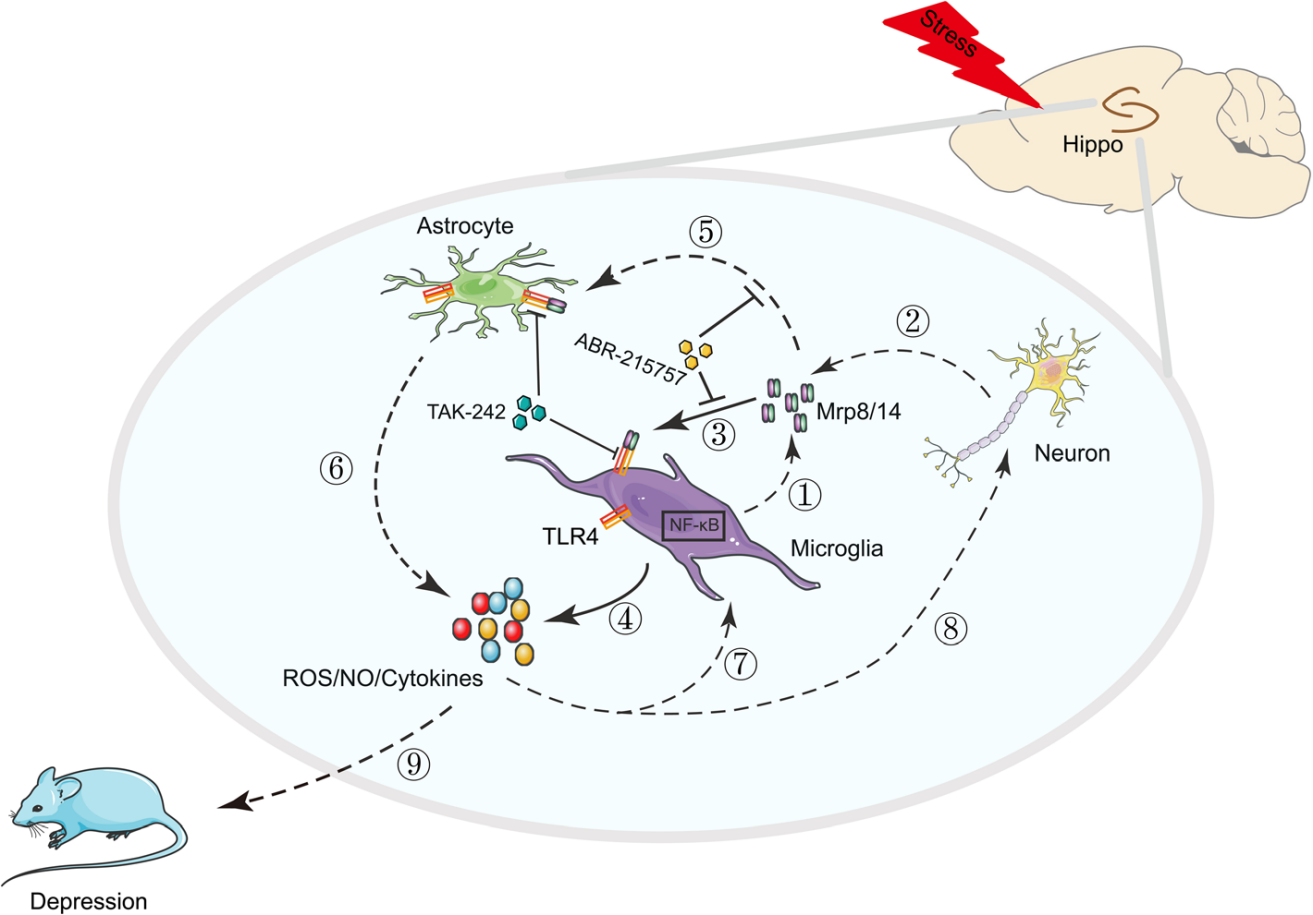
Proposed working model for Mrp8/14 in the development of depressive symptoms. CUMS, chronic unpredictable mild stress; Mrp8/14, myeloid-related protein-8/14; ABR-215757, N-ethyl-N-phenyl-1,2-dihydro-5-ethyl-4-hydroxyl-1-mehtyl-2-oxo-quinoline-3-carboxamide; TLR4, Toll-like receptor-4; TAK-242, ethyl (6R)-6-[N-(2-Chloro-4-fluorophenyl) sulfamoyl] cyclohex-1-ene-1-carboxylate; Hippo, hippocampus; NF-κB, nuclear factor-kappa B; ROS, reactive oxygen species; NO, nitric oxide

Clinical and animal studies suggest that neuroinflammation is a key component of depressive symptoms [3, 4, 40, 41]. As DAMPs, Mrp8 and its binding partner Mrp14 are upregulated after chronic stress exposure [26] and impact numerous inflammatory diseases [42]. Our findings showed that Mrp8 and Mrp14 increased with depressive-like behaviors in CUMS-treated mice. Pharmacological inhibition of Mrp14 attenuated CUMS-induced TLR4/NF-κB signaling activation and depressive-like behaviors. Central injection of these recombinant proteins could evoke depressive-like behaviors, TLR4/NF-κB signaling activation as well as microglia activation. Effects of recombinant Mrp8/14 were attenuated by TLR4 inhibitor TAK-242. Therefore, these findings suggest that the dysfunction of Mrp8/14-TLR4 signaling in the hippocampus can result in neuroinflammation and depressive-like behaviors (Fig. 7).

We conducted the present study focusing on the hippocampus, which is a key brain region in the development of depression [43, 44]. As expected, four-week CUMS provoked depressive-like behaviors, including decreased sucrose preference and increased immobility time in TST. A forced swimming test is not adopted for examining the behavioral change because immobility in this test is adaptive and does not reflect depressive symptoms [45]. We found both Mrp8 and Mrp14 were increased in the hippocampus and serum of stressed mice. This finding matches up with the previous report of chronic stress that upregulated Mrp8 and Mrp14 mRNA [26]. According to previous studies [20, 22], excessive Mrp8 and Mrp14 may be released from hippocampal neurons and microglia. Thus, extracellular Mrp8/14 can further affect other adjacent astrocytes and microglia. Indeed, extracellular Mrp8/14 can potentiate astrocyte and microglia activation, and then, directly and indirectly affect neurons [21, 23]. Although it is not entirely clear whether intracellular homodimers of Mrp8 and Mrp14 exist, the heterodimeric complex of Mrp8/14 is the most abundant form and seems to be indispensable [13]. Unfortunately, no commercial ELISA kit or antibody is provided for specifically detecting Mrp8/14 heterodimer in rodents. We tried to make up for this limitation by administrating recombinant Mrp8/14 heterodimer in the following experiments. Future studies focusing on the formation mechanism of Mrp8/14 heterodimer and the functional difference between the monomer and heterodimer are urgently needed.

To fulfill its proposed function as an endogenous DAMP protein, extracellular Mrp8/14 can be recognized by pattern recognition receptors (PRRs), such as TLR4 and RAGE [13]. As reported in a previous study [34], TLR4/NF-κB signaling was activated in CUMS-exposed mice. Besides, no significant differences were found in RAGE receptor expression between control mice and stressed mice. In contrast to our results, a previous study demonstrated chronic mild stress (CMS) significantly downregulated hippocampal RAGE protein [46]. The reasons for the discrepancy are unclear, but the different species and stress protocol may be two possible reasons. In the present study, we adopted four-week unpredictable CMS using BALB/c mice at 6–8 weeks of age, while the other study performed three-week CMS using Sprague–Dawley rats at 5–7 weeks of age. RAGE receptor may change differently between mice and rats. Furthermore, the effects of stress may be alleviated because the rats may adapt to the stressor. This is why we choose unpredictable CMS protocol. Different stress intensity may result in different responses of RAGE receptor.

In Mrp14^−/−^ mice, Mrp8 is not expressed, but the transcription of Mrp8 is not altered [13]. Thus, Mrp8 may be influenced by Mrp14 on the protein level. The FDA approved drug, ABR-215757, has been reported as an inhibitor of Mrp14. ABR-215757 contributes to the reduction of inflammatory response by blocking the interaction with TLR4 and RAGE [47]. Our results verified that expression and activation of hippocampal TLR4 signaling were suppressed by ABR-215757 compound, while ABR-215757 did not affect the expression of RAGE. Downstream TLR4/NF-κB signaling pathway was not determined in the present study since plenty of evidence has indicated the activation of TLR4/NF-κB signaling pathway in stress-induced neuroinflammation [34, 48]. As a result of TLR4 signaling blocking, the depressive-like behaviors were successfully rescued by ABR-215757 treatment. These results suggest that CUMS-provoked depressive-like behaviors are mediated by hippocampal Mrp14 or Mrp8/14.

Furthermore, recombinant Mrp8, Mrp14, and Mrp8/14 heterodimer were administrated by ICV injection to detect their effects on behaviors and the underlying mechanism. The results showed that depressive-like behaviors and TLR4/NF-κB signaling activation were observed in all the three protein-administrated mice. However, we did not find any effects of these recombinant proteins on RAGE expression. These findings are in line with our previous results using a CUMS animal model. It should be noted that the recombinant proteins were used at high doses in this study. Central injection of these recombinants at lower doses may not induce neuroinflammation and depressive-like behaviors in mice.

Mrp8/14 can induce the release of IL-6, IL-8, IL-1β, and TNF-α in monocytes or bone marrow cells [9, 49]. As the primary immune cells of the CNS, primed microglia act as the major contributors to neuroinflammation [50]. Microglial alterations contribute to the development of depressive-like behaviors [37], and minocycline (an inhibitor of microglia activation) treatment ameliorates depressive-like behaviors in rodents [51, 52]. Even, to some extent, depression can be considered as a microglial disease (microgliopathy) [36]. Therefore, we also tested microglial alternation and the subsequent neuroinflammation. All of the three recombinant proteins could induce the overexpression of IBA-1 and proinflammatory cytokines (TNF-α, IL-1β, IL-6) in hippocampus associated with the depressive-like behaviors. This is partly supported by another report, which indicates that Mrp8 induces hippocampal microglia activation and exerts proinflammatory effects in a tibial fracture surgery mice model [32].

Next, we further verified whether the recombinant proteins-induced depressive-like behaviors were mediated by TLR4 signaling and microglia activation. Although all of Mrp8, Mrp14, and Mrp8/14 seem to be effective, we choose Mrp8/14 heterodimer for this issue. The main reason is that the heterodimer is the most abundant form [11, 12]. Besides, Mrp8 or Mrp14 may also have effects by binding the other partner and forming Mrp8/14 heterodimer. The results demonstrated that TLR4 inhibitor TAK-242 could attenuate Mrp8/14-induced depressive-like behaviors and the upregulation of proinflammatory cytokines. Thus, Mrp8/14 may have effects via modulating TLR4 signaling pathway. Here, our results did not include a group of TAK-242 as our previous study has indicated that TAK-242 administration does not affect the behavioral consequences compared to the control group [6]. To minimize the use of animals, we did not assign the group of TAK-242 alone administration. It should be noted that RAGE may also be involved in the behavioral and biological changes induced by Mrp8/14 despite its expression is unchanged. Recently, Franklin and colleagues found that microglial RAGE contributed to chronic stress-induced priming of depressive-like behavior [53]. Another in vitro study indeed has demonstrated that RAGE but not TLR4 associates with Mrp8/14 in colon tumor cells [54]. This issue could be addressed in future experiments by using RAGE knockout animals in various models of depressive symptoms.

As two indicators of microglia activation, NO and ROS have been suggested to contribute to the development of depressive symptoms [55–58]. iNOS-mediated NO synthesis and NOX1/NADPH oxidase-mediated ROS generation play crucial roles in the pathophysiological processes of depressive-like behaviors [55–57]. On the other hand, microglia activation may depend on TLR4 signaling in diverse animal models [59, 60]. Our results showed the ROS- and iNOS-mediated NO generations were markedly enhanced after Mrp8/14 treatment in BV2 microglia. The inhibition of TLR4 attenuated these effects of Mrp8/14. These results suggest that Mrp8/14-induced microglia activation depends on TLR4 signaling. The generation of NO and ROS derived from activated microglia may promote the depressive symptoms. The inhibition of TLR4 may provide beneficial antidepressant effects via suppressing microglia activation. Moreover, the products (NO, ROS, and inflammatory cytokines) from activated microglia may affect neurons and amplify neuroinflammation (Fig. 7).

## Conclusions

In conclusion, we identify a vital molecule contributing to the development of depressive symptoms and augment neuroinflammation. Our results validate that Mrp8/14 takes a critical role in CUMS-provoked neuroinflammation and depressive-like behaviors. The Mrp14 inhibitor ABR-215757 effectively ameliorates depressive symptoms and TLR4/NF-κB signaling activation. Central injection of bioactive recombinant protein confirms the role of Mrp8/14 in proinflammatory cytokines overexpression and the development of depressive-like behaviors. These Mrp8/14-induced cellular, biochemical, and behavioral changes depend on TLR4 signaling. Our results further reinforce the neuroinflammation hypothesis of depression. These findings also provide new sights into the underlying molecular mechanism of depression and raise a novel antidepressant approach by targeting the aberrant Mrp8/14 function.

## Additional files

Additional file 1: **Figure S1.** The schematic diagrams showing the experimental designs. CUMS, chronic unpredictable mild stress; SPT, sucrose preference test; TST, tail suspension test; ICV cannulation, intracerebroventricular cannulation; IP, intraperitoneal. (TIF 1686 kb)

Additional file 2: **Table S1.** Primer sequence used in this study. (DOCX 17 kb)

## Abbreviations

ATCC: American type culture collection
ATP: Adenosine triphosphate
CMS: Chronic mild stress
CNS: Central nervous systems
CUMS: Chronic unpredictable mild stress
DAMPs: Damage-associated molecular patterns
ELISA: Enzyme-linked immunosorbent assay kits
HMGB1: High mobility group box 1
ICV: Intracerebroventricular
IP: Intraperitoneal
Mrp: Myeloid-related protein
NO: Nitric oxide
PRRs: Pattern recognition receptors
RAGE: Receptor for advanced glycation
ROS: Reactive oxygen species
SEM: Standard error of the mean
SLE: Systemic lupus erythematosus
SPT: Sucrose preference test
TLR4: Toll-like receptor 4
TST: Tail suspension test

## References

[1] Mueller TI, Leon AC, Keller MB, Solomon DA, Endicott J, Coryell W, Warshaw M, Maser JD (1999). Recurrence after recovery from major depressive disorder during 15 years of observational follow-up. Am J Psychiatry.

[2] Connolly KR, Thase ME (2012). Emerging drugs for major depressive disorder. Expert Opin Emerg Drugs.

[3] Miller AH, Raison CL (2016). The role of inflammation in depression: from evolutionary imperative to modern treatment target. Nat Rev Immunol.

[4] Wohleb ES, Franklin T, Iwata M, Duman RS (2016). Integrating neuroimmune systems in the neurobiology of depression. Nat Rev Neurosci.

[5] Wu TY, Liu L, Zhang W, Zhang Y, Liu YZ, Shen XL, Gong H, Yang YY, Bi XY, Jiang CL, Wang YX (2015). High-mobility group box-1 was released actively and involved in LPS induced depressive-like behavior. J Psychiatr Res.

[6] Lian YJ, Gong H, Wu TY, Su WJ, Zhang Y, Yang YY, Peng W, Zhang T, Zhou JR, Jiang CL, Wang YX (2017). Ds-HMGB1 and fr-HMGB induce depressive behavior through neuroinflammation in contrast to nonoxid-HMGB1. Brain Behav Immun.

[7] Wang B, Lian YJ, Su WJ, Peng W, Dong X, Liu LL, Gong H, Zhang T, Jiang CL, Wang YX. HMGB1 mediates depressive behavior induced by chronic stress through activating the kynurenine pathway. Brain Behav Immun. 2017;72:51–60. 10.1016/j.bbi.2017.11.017 29195782

[8] Cao X, Li LP, Wang Q, Wu Q, Hu HH, Zhang M, Fang YY, Zhang J, Li SJ, Xiong WC (2013). Astrocyte-derived ATP modulates depressive-like behaviors. Nat Med.

[9] Vogl T, Tenbrock K, Ludwig S, Leukert N, Ehrhardt C, van Zoelen MA, Nacken W, Foell D, van der Poll T, Sorg C, Roth J (2007). Mrp8 and Mrp14 are endogenous activators of Toll-like receptor 4, promoting lethal, endotoxin-induced shock. Nat Med.

[10] Ehrchen JM, Sunderkotter C, Foell D, Vogl T, Roth J (2009). The endogenous Toll-like receptor 4 agonist S100A8/S100A9 (calprotectin) as innate amplifier of infection, autoimmunity, and cancer. J Leukoc Biol.

[11] Hunter MJ, Chazin WJ (1998). High level expression and dimer characterization of the S100 EF-hand proteins, migration inhibitory factor-related proteins 8 and 14. J Biol Chem.

[12] Vogl T, Gharibyan AL, Morozova-Roche LA (2012). Pro-inflammatory S100A8 and S100A9 proteins: self-assembly into multifunctional native and amyloid complexes. Int J Mol Sci.

[13] Pruenster M, Vogl T, Roth J, Sperandio M (2016). S100A8/A9: from basic science to clinical application. Pharmacol Ther.

[14] Schonthaler HB, Guinea-Viniegra J, Wculek SK, Ruppen I, Ximenez-Embun P, Guio-Carrion A, Navarro R, Hogg N, Ashman K, Wagner EF (2013). S100A8-S100A9 protein complex mediates psoriasis by regulating the expression of complement factor C3. Immunity.

[15] Bengtsson AA, Sturfelt G, Lood C, Ronnblom L, van Vollenhoven RF, Axelsson B, Sparre B, Tuvesson H, Ohman MW, Leanderson T (2012). Pharmacokinetics, tolerability, and preliminary efficacy of paquinimod (ABR-215757), a new quinoline-3-carboxamide derivative: studies in lupus-prone mice and a multicenter, randomized, double-blind, placebo-controlled, repeat-dose, dose-ranging study in **patients with systemic lupus erythematosus**. Arthritis Rheum.

[16] Hurnakova J, Zavada J, Hanova P, Hulejova H, Klein M, Mann H, Sleglova O, Olejarova M, Forejtova S, Ruzickova O (2015). Serum calprotectin (S100A8/9): an independent predictor of ultrasound synovitis in patients with rheumatoid arthritis. Arthritis Res Ther.

[17] Nagareddy PR, Kraakman M, Masters SL, Stirzaker RA, Gorman DJ, Grant RW, Dragoljevic D, Hong ES, Abdel-Latif A, Smyth SS (2014). Adipose tissue macrophages promote myelopoiesis and monocytosis in obesity. Cell Metab.

[18] Jin GZ, Dong W, Dong H, Yu H, Chen J, Yu WL, Li AJ, Cong WM, Wu MC (2015). The diagnostic and prognostic value of MRP8/MRP14 in intrahepatic cholangiocarcinoma. Oncotarget.

[19] Zhang X, Ai F, Li X, She X, Li N, Tang A, Qin Z, Ye Q, Tian L, Li G (2015). Inflammation-induced S100A8 activates Id3 and promotes colorectal tumorigenesis. Int J Cancer.

[20] Kummer MP, Vogl T, Axt D, Griep A, Vieira-Saecker A, Jessen F, Gelpi E, Roth J, Heneka MT (2012). Mrp14 deficiency ameliorates amyloid beta burden by increasing microglial phagocytosis and modulation of amyloid precursor protein processing. J Neurosci.

[21] Wang C, Klechikov AG, Gharibyan AL, Warmlander SK, Jarvet J, Zhao L, Jia X, Narayana VK, Shankar SK, Olofsson A (2014). The role of pro-inflammatory S100A9 in Alzheimer's disease amyloid-neuroinflammatory cascade. Acta Neuropathol.

[22] Ryu MJ, Liu Y, Zhong X, Du J, Peterson N, Kong G, Li H, Wang J, Salamat S, Chang Q, Zhang J (2012). Oncogenic KRAS expression in postmitotic neurons leads to S100A8-S100A9 protein overexpression and gliosis. J Biol Chem.

[23] Gan N, Yang L, Omran A, Peng J, Wu L, He F, Zhang C, Xiang Q, Kong H, Ma Y (2014). Myoloid-related protein 8, an endogenous ligand of Toll-like receptor 4, is involved in epileptogenesis of mesial temporal lobe epilepsy via activation of the nuclear factor-kappaB pathway in astrocytes. Mol Neurobiol.

[24] Wache C, Klein M, Ostergaard C, Angele B, Hacker H, Pfister HW, Pruenster M, Sperandio M, Leanderson T, Roth J (2015). Myeloid-related protein 14 promotes inflammation and injury in meningitis. J Infect Dis.

[25] Ziegler G, Prinz V, Albrecht MW, Harhausen D, Khojasteh U, Nacken W, Endres M, Dirnagl U, Nietfeld W, Trendelenburg G (2009). Mrp-8 and -14 mediate CNS injury in focal cerebral ischemia. Biochim Biophys Acta.

[26] Stankiewicz AM, Goscik J, Majewska A, Swiergiel AH, Juszczak GR (2015). The effect of acute and chronic social stress on the hippocampal transcriptome in mice. PLoS One.

[27] Hill MN, Hellemans KG, Verma P, Gorzalka BB, Weinberg J (2012). Neurobiology of chronic mild stress: parallels to major depression. Neurosci Biobehav Rev.

[28] Peng YL, Liu YN, Liu L, Wang X, Jiang CL, Wang YX (2012). Inducible nitric oxide synthase is involved in the modulation of depressive behaviors induced by unpredictable chronic mild stress. J Neuroinflammation.

[29] Iwata M, Ota KT, Li XY, Sakaue F, Li N, Dutheil S, Banasr M, Duric V, Yamanashi T, Kaneko K (2016). Psychological stress activates the inflammasome via release of adenosine triphosphate and stimulation of the purinergic type 2X7 receptor. Biol Psychiatry.

[30] Zhang Y, Liu L, Liu YZ, Shen XL, Wu TY, Zhang T, Wang W, Wang YX, Jiang CL. NLRP3 inflammasome mediates chronic mild stress-induced depression in mice via neuroinflammation. Int J Neuropsychopharmacol. 2015;18. 10.1093/ijnp/pyv006. 10.1093/ijnp/pyv006 PMC4571628 25603858

[31] Yan L, Bjork P, Butuc R, Gawdzik J, Earley J, Kim G, Hofmann Bowman MA (2013). Beneficial effects of quinoline-3-carboxamide (ABR-215757) on atherosclerotic plaque morphology in S100A12 transgenic ApoE null mice. Atherosclerosis.

[32] Lu SM, Yu CJ, Liu YH, Dong HQ, Zhang X, Zhang SS, Hu LQ, Zhang F, Qian YN, Gui B (2015). S100A8 contributes to postoperative cognitive dysfunction in mice undergoing tibial fracture surgery by activating the TLR4/MyD88 pathway. Brain Behav Immun.

[33] Hua F, Tang H, Wang J, Prunty MC, Hua X, Sayeed I, Stein DG (2015). TAK-242, an antagonist for Toll-like receptor 4, protects against acute cerebral ischemia/reperfusion injury in mice. J Cereb Blood Flow Metab.

[34] Garate I, Garcia-Bueno B, Madrigal JL, Caso JR, Alou L, Gomez-Lus ML, Mico JA, Leza JC (2013). Stress-induced neuroinflammation: role of the Toll-like receptor-4 pathway. Biol Psychiatry.

[35] Hung YY, Lin CC, Kang HY, Huang TL (2017). TNFAIP3, a negative regulator of the TLR signaling pathway, is a potential predictive biomarker of response to antidepressant treatment in major depressive disorder. Brain Behav Immun.

[36] Yirmiya R, Rimmerman N, Reshef R (2015). Depression as a microglial disease. Trends Neurosci.

[37] Kreisel T, Frank MG, Licht T, Reshef R, Ben-Menachem-Zidon O, Baratta MV, Maier SF, Yirmiya R (2014). Dynamic microglial alterations underlie stress-induced depressive-like behavior and suppressed neurogenesis. Mol Psychiatry.

[38] Ghasemi M, Fatemi A (2014). Pathologic role of glial nitric oxide in adult and pediatric neuroinflammatory diseases. Neurosci Biobehav Rev.

[39] Qiu LL, Ji MH, Zhang H, Yang JJ, Sun XR, Tang H, Wang J, Liu WX, Yang JJ (2016). NADPH oxidase 2-derived reactive oxygen species in the hippocampus might contribute to microglial activation in postoperative cognitive dysfunction in aged mice. Brain Behav Immun.

[40] Najjar S, Pearlman DM, Hirsch S, Friedman K, Strange J, Reidy J, Khoukaz M, Ferrell RB, Devinsky O, Najjar A, Zagzag D (2014). Brain biopsy findings link major depressive disorder to neuroinflammation, oxidative stress, and neurovascular dysfunction: a case report. Biol Psychiatry.

[41] Dobos N, Korf J, Luiten PG, Eisel UL (2010). Neuroinflammation in Alzheimer's disease and major depression. Biol Psychiatry.

[42] Roth J, Vogl T, Sorg C, Sunderkotter C (2003). Phagocyte-specific S100 proteins: a novel group of proinflammatory molecules. Trends Immunol.

[43] Mahar I, Bambico FR, Mechawar N, Nobrega JN (2014). Stress, serotonin, and hippocampal neurogenesis in relation to depression and antidepressant effects. Neurosci Biobehav Rev.

[44] Geerlings MI, Gerritsen L. Late-life depression, hippocampal volumes, and hypothalamic-pituitary-adrenal axis regulation: a systematic review and meta-analysis. Biol Psychiatry. 2017;82:339-50. 10.1016/j.biopsych.2016.12.032 28318491

[45] Molendijk ML, de Kloet ER (2015). Immobility in the forced swim test is adaptive and does not reflect depression. Psychoneuroendocrinology.

[46] Rong H, Wang G, Liu T, Wang H, Wan Q, Weng S (2010). Chronic mild stress induces fluoxetine-reversible decreases in hippocampal and cerebrospinal fluid levels of the neurotrophic factor S100B and its specific receptor. Int J Mol Sci.

[47] Bjork P, Bjork A, Vogl T, Stenstrom M, Liberg D, Olsson A, Roth J, Ivars F, Leanderson T (2009). Identification of human S100A9 as a novel target for treatment of autoimmune disease via binding to quinoline-3-carboxamides. PLoS Biol.

[48] Garate I, Garcia-Bueno B, Madrigal JL, Caso JR, Alou L, Gomez-Lus ML, Leza JC (2014). Toll-like 4 receptor inhibitor TAK-242 decreases neuroinflammation in rat brain frontal cortex after stress. J Neuroinflammation.

[49] Fassl SK, Austermann J, Papantonopoulou O, Riemenschneider M, Xue J, Bertheloot D, Freise N, Spiekermann C, Witten A, Viemann D (2015). Transcriptome assessment reveals a dominant role for TLR4 in the activation of human monocytes by the alarmin MRP8. J Immunol.

[50] Fenn AM, Gensel JC, Huang Y, Popovich PG, Lifshitz J, Godbout JP (2014). Immune activation promotes depression 1 month after diffuse brain injury: a role for primed microglia. Biol Psychiatry.

[51] Wang HT, Huang FL, Hu ZL, Zhang WJ, Qiao XQ, Huang YQ, Dai RP, Li F, Li CQ. Early-life social isolation-induced depressive-like behavior in rats results in microglial activation and neuronal histone methylation that are mitigated by minocycline. Neurotox Res. 2017;31:505–20. 10.1007/s12640-016-9696-3 28092020

[52] Zheng LS, Kaneko N, Sawamoto K (2015). Minocycline treatment ameliorates interferon-alpha- induced neurogenic defects and depression-like behaviors in mice. Front Cell Neurosci.

[53] Franklin TC, Wohleb ES, Zhang Y, Fogaca M, Hare B, Duman RS. Persistent increase in microglial RAGE contributes to chronic stress-induced priming of depressive-like behavior. Biol Psychiatry. 2018;83:50–60. 10.1016/j.biopsych.2017.06.034 PMC6369917 28882317

[54] Hermani A, De Servi B, Medunjanin S, Tessier PA, Mayer D (2006). S100A8 and S100A9 activate MAP kinase and NF-kappaB signaling pathways and trigger translocation of RAGE in human prostate cancer cells. Exp Cell Res.

[55] Seo JS, Park JY, Choi J, Kim TK, Shin JH, Lee JK, Han PL (2012). NADPH oxidase mediates depressive behavior induced by chronic stress in mice. J Neurosci.

[56] Ibi M, Liu J, Arakawa N, Kitaoka S, Kawaji A, Matsuda KI, Iwata K, Matsumoto M, Katsuyama M, Zhu K, et al. Depressive-like behaviors are regulated by NOX1/NADPH oxidase by redox modification of NMDA receptor 1. J Neurosci. 2017;37:4200-12. 10.1523/JNEUROSCI.2988-16.2017 PMC6596584 28314819

[57] Montezuma K, Biojone C, Lisboa SF, Cunha FQ, Guimaraes FS, Joca SR (2012). Inhibition of iNOS induces antidepressant-like effects in mice: pharmacological and genetic evidence. Neuropharmacology.

[58] Filipovic D, Todorovic N, Bernardi RE, Gass P (2017). Oxidative and nitrosative stress pathways in the brain of socially isolated adult male rats demonstrating depressive- and anxiety-like symptoms. Brain Struct Funct.

[59] Papageorgiou IE, Lewen A, Galow LV, Cesetti T, Scheffel J, Regen T, Hanisch UK, Kann O (2016). TLR4-activated microglia require IFN-gamma to induce severe neuronal dysfunction and death in situ. Proc Natl Acad Sci U S A.

[60] Bell MT, Puskas F, Agoston VA, Cleveland JC, Freeman KA, Gamboni F, Herson PS, Meng X, Smith PD, Weyant MJ (2013). Toll-like receptor 4-dependent microglial activation mediates spinal cord ischemia-reperfusion injury. Circulation.

