# Supplementary figures and images for: Hippocampal Mrp8/14 signaling plays a critical role in the manifestation of depressive-like behaviors in mice
Source: J Neuroinflammation. 2018 Sep 4;15:252. doi: 10.1186/s12974-018-1296-0 (PMC6122683; doi:10.1186/s12974-018-1296-0)

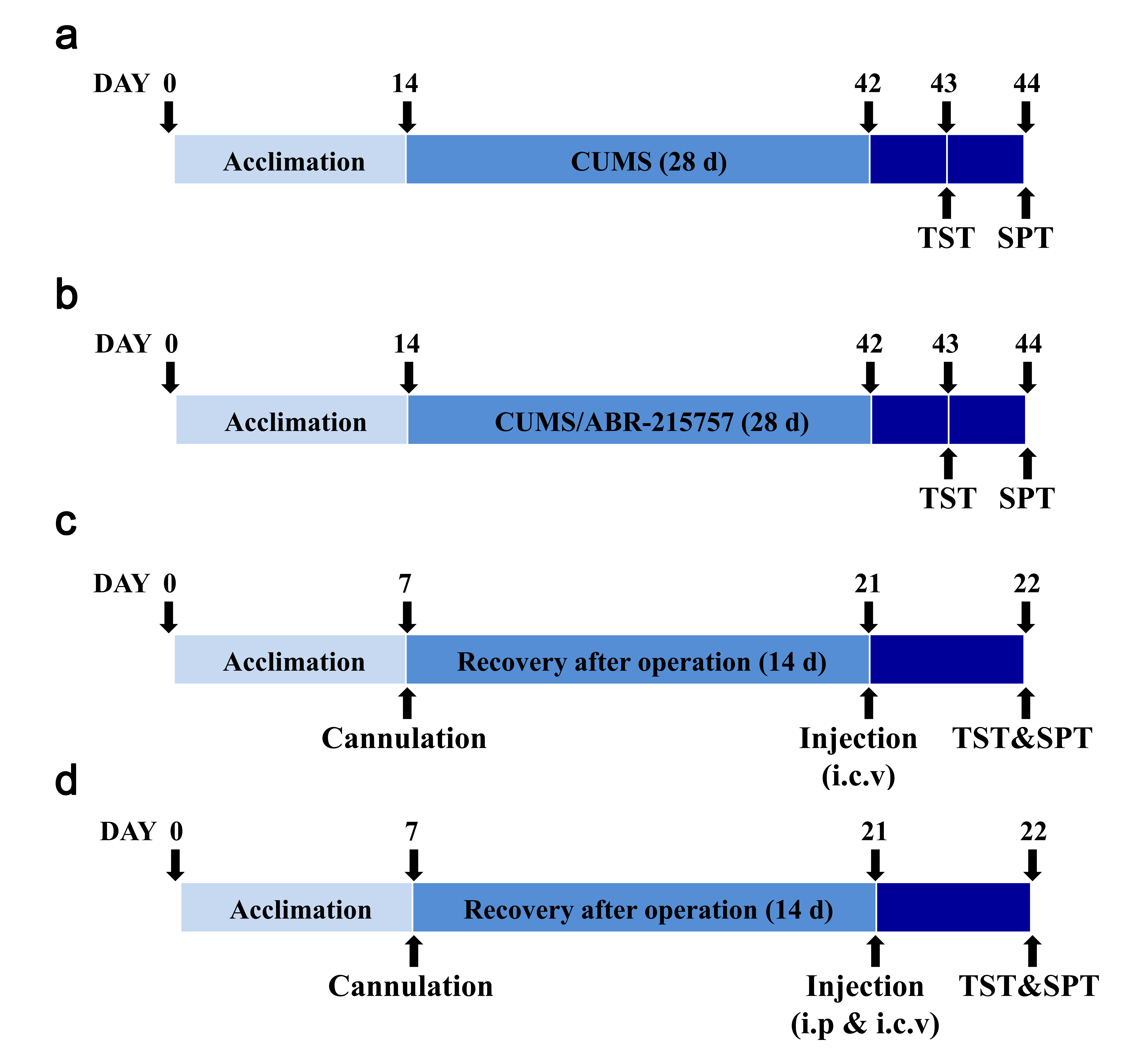

Supplement: Supplementary file 1 — Figure S1. The schematic diagrams showing the experimental designs. CUMS, chronic unpredictable mild stress; SPT, sucrose preference test; TST, tail suspension test; ICV cannulation, intracerebroventricular cannulation; IP, intraperitoneal. (TIF 1686 kb) [file 12974_2018_1296_MOESM1_ESM.tif]
