# Supplementary material for: Hippocampal Mrp8/14 signaling plays a critical role in the manifestation of depressive-like behaviors in mice
Source: J Neuroinflammation. 2018 Sep 4;15:252. doi: 10.1186/s12974-018-1296-0 (PMC6122683; doi:10.1186/s12974-018-1296-0)
Supplement: Supplementary file 2 — Table S1. Primer sequence used in this study. (DOCX 17 kb) [file 12974_2018_1296_MOESM2_ESM.docx]

**Table S1** Primer sequence used in this study.

| **Gene** | **Primer sequence 5’→3’** |
| --- | --- |
| IBA-1 | Forward: ATCAACAAGCAATTCCTCGATGA  Reverse: CAGCATTCGCTTCAAGGACATA |
| TNFα | Forward: TGTCTCAGCCTCTTCTCATTCC  Reverse: TGAGGGTCTGGGCCATAGAAC |
| IL-1β | Forward: CTGTGACTCATGGGATGATGATG  Reverse: CGGAGCCTGTAGTGCAGTTG |
| IL-6 | Forward: TAGTCCTTCCTACCCCAATTTCC  Reverse: TTGGTCCTTAGCCACTCCTTC |
| iNOS | Forward: GTTCTCAGCCCAACAATACAAGA  Reverse: GTGGACGGGTCGATGTCAC |
| β-Actin | Forward: TTCTTGGGTATGGAATCCTGT  Reverse: AGCACTGTGTTGGCATAGAG |
